# Splitting tensile strength and microstructure of xanthan gum-treated loess

**DOI:** 10.1038/s41598-022-14058-4

**Published:** 2022-06-15

**Authors:** Tong Jiang, Jin-di Zhao, Jun-ran Zhang

**Affiliations:** School of Earth Sciences and Engineering, North China University of Water Conservancy and Electric Power, Zhengzhou, 450046 Henan China

**Keywords:** Ecology, Environmental sciences, Natural hazards, Engineering

## Abstract

The tensile strength of loess is closely related to geological disasters. As eco-friendly materials, biopolymers have an excellent strengthening effect on the mechanical properties of soil. The effect of different initial dry densities and xanthan gum (XG) contents on the microstructure and mechanical behavior of XG-treated loess was studied with a series of microscopic tests and splitting tensile tests based on the particle image velocimetry technique. The results show that the XG became concentrated and agglomerated during dehydration, forming bridge links between soil particles and covering their surfaces. The XG-treated loess had a significant concentration of micropores and mesopores, with greater peak pore size distribution values than untreated loess. The specimens’ load–displacement curves with different XG contents and initial dry densities showed strain-softening. The displacement vector field indicated that specimens’ primary cracks were radial–vertical, and the secondary cracks were well-developed. The strain-softening phenomenon was more significant with increased XG content and initial dry density, and the specimens’ splitting tensile strength and brittleness increased. XG treatment gave the soils stronger cementation and a denser structure, helping to increase strength and brittleness. This research provides a scientific basis and practical experience for applying XG in geotechnical engineering.

## Introduction

Loess has high silt content, low natural water content, high porosity, low natural tensile strength, and readily collapses when exposed to water. It is easy to produce a differential settlement, cracking, and collapse when directly used as engineering filler, making it challenging to meet actual construction requirements^1,2^. As one of the three significant soil strengths, tensile strength is often neglected because of soil’s relatively weak tensile resistance. However, geological disasters and engineering accidents such as ground cracks, landslides, earth-rock dam cracking, and slope instability are related to the low tensile strength of loess^3–6^. Therefore, reinforcing loess and studying its modification of tensile properties is of great practical significance.

Cement, lime, and fly ash have been widely used in engineering construction in loess areas. The geotechnical properties of soil can be improved by these modified materials either by single addition or combined incorporation^7,8^. Nevertheless, the production of these materials is accompanied by large amounts of dust and wastewater, polluting the environment and harming human health. With calls for national green environmental protection, many techniques to stabilize loess have emerged. Root, wheat straw, lignin fiber, fiber yarn, glass fiber, polypropylene fiber, and basalt fiber have been used as modified materials for soil stabilization^9–14^. The improvement of loess engineering properties using stabilizing agents such as F1, HEC, SH, and nano-SiO_2_ has also been explored^15–18^. Liu et al.^19^ pointed out that microbial-induced carbonate precipitation (MICP) was widely used in civil engineering. However, these technologies are undesirable for various engineering practices because of their high cost, stringent environmental requirements, and the complete dependence on material strength improvements^20^. Therefore, it is necessary to explore economic and eco-friendly soil treatment techniques.

As a renewable and cost-effective material, existing studies have shown that biopolymers have excellent performance in soil reinforcement, promoting vegetation growth and desert control^21–23^. As one of the biopolymers, xanthan gum (XG) possesses high viscosity even at very low concentrations and exhibits strong hydrophilicity, high pseudo-plasticity, and high tolerance to acids, alkalis, salt, and heat. These advantages create conditions for its wide application as a food binder, biomedicine, and petroleum exploitation^24–26^. XG also shows strong potential for soil stabilization. Kumar et al.^27^ showed that XG could provide higher unconfined compressive strength than guar gum and β-glucan. Chang et al.^28^ pointed out that adding a small amount of XG (1.0%) could enhance soil erosion resistance. Compared with a wet mixture, dry mixing can achieve higher material strength. Fatehi et al.^29^ stated that when the XG content exceeded 2.0%, the strength of XG-treated soil tended to decline. Lee et al.^30,31^ modified sand with XG and found that the shear strength of the specimens most improved under dehydrated XG conditions. Chang et al.^32^ proposed that the entire interaction between XG and soil particles could be realized at low water content. Loess exists widely in a semiarid to arid climate with low natural water content, providing suitable conditions for XG application to improve loess properties. Notably, the feasibility of biopolymer-solidified loess has been validated. For example, Liu et al.^33^ found that polymer treatment significantly controlled soil sheet erosion of loess slopes. Pu et al.^34^ showed that polymers could reinforce loess strength. Zhang et al.^35^ indicated that polymers could improve the anti-disintegration property and water retention capacity of loess. In addition, XG could increase the fatigue life of soil^36^ and reduce the rheological properties, permeability, and dispersion of soil^37–41^.

In tensile test research, Muguda et al.^42,43^ proved through a direct tensile test that all XG-treated soil has higher tensile strength than untreated soil at seven days. Soldo et al.^44^ carried out splitting tests and showed that XG had more significant soil tensile strength improvement than other biopolymers. However, there are few studies on the influence of XG on the tensile strength of loess. The radial splitting tensile test can measure soil tensile strength indirectly and is selected in this paper because it is a rapid and straightforward technique^45,46^.

One way to explain a material’s mechanical behavior is to study its microstructure. During drying, XG enhances soil strength by coating the particles’ surfaces, improves permeability by filling pores, and improves erosion resistance by increasing fluid viscosity^47–50^. In addition, the ability of soil to resist external deformation under stress is enhanced by cation bridging and hydrogen bonds between XG’s carboxylic acid (-COOH) and hydroxyl (-OH) groups and the cations on the particle surfaces^51^. Scanning electron microscopy (SEM) is currently the primary method used for microscopic analysis. However, mercury intrusion porosimetry (MIP), as a quantitative way to describe the porosity and pore size distribution of soil, is rarely used in the analysis of polymer-treated soils.

In support of the Sanmenxia section construction phase of the China National Highway G310 project, a series of splitting tension tests were conducted using a particle image velocimetry (PIV) test system with different initial dry densities (1.55 g/cm^3^, 1.63 g/cm^3^, and 1.70 g/cm^3^) and XG contents (0.0%, 0.5%, 1.0%, and 2.0%). In addition, SEM and MIP tests were used to conduct qualitative and quantitative analyses on the microstructure of XG-treated loess, which helped to further explain XG-treated loess macroscopic behavior. The results will provide a reference for theoretical research and engineering application of XG-treated loess.

## Materials

**Figure 1:**
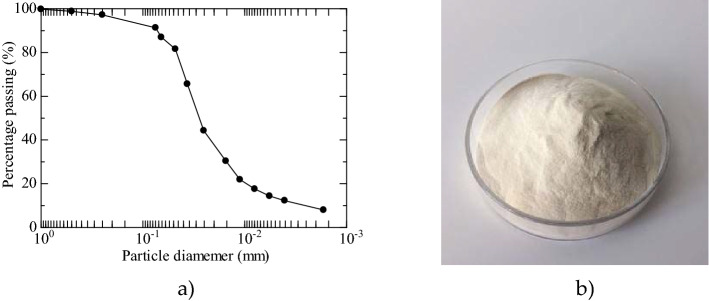
(**a**) Loess particle-size distribution; (**b**) Xanthan gum (XG) powder.

The loess was selected from near the bridge of Dongshang Village, Lingbao City, Sanmenxia in Henan Province, China. The essential physical characteristics of this loess are shown in Table 1. According to the grain size distribution curve in Fig. 1a, the loess was 77.24% silt, 14.10% clay, and 8.66% sand. It had a coefficient of uniformity (*C*_u_) of 2.57 and a coefficient of curvature (*C*_c_) of 12.43. Other basic physical parameters are shown in Table 1. According to the engineering classification standard, the loess can be classified as well-graded silty soil with low plasticity.

**Table 1:** Loess physical parameters.

Physical parameters	Values
Liquid limit, *w*_L_ (%)	26.92
Plastic limit, *w*_P_ (%)	18.06
Plasticity index, *I*_P_	8.86
Specific gravity of the solid, *G*_s_	2.70
Natural water content, *w*_0_ (%)	5.10
Natural dry weight, *ρ*_d0_ (g/cm^3^)	1.33
Optimal water content, *w*_opt_ (%)	17.70
Maximum dry density, *ρ*_dmax_ (g/cm^3^)	1.74

The xanthan gum (XG) used in the test is a white to cream-colored powder, as shown in Fig. 1b. It is an anionic extracellular branching polysaccharide produced by the bacterium *Xanthomonas campestris* and is economical and widely available. XG mainly forms viscous hydrogels with a primary structure consisting of two glucose, two mannoses, and one glucuronic acid. The parameters for XG are shown in Table 2.

**Table 2:** Xanthan gum (XG) parameters.

Biopolymer	Grade	Viscosity (cP)	Pyruvic acid (%)	Dry weight loss (%)
XG	Food	1475	≥ 1.5	9.0

### Test apparatus and experimental techniques

#### Splitting tension test equipment based on PIV technology

**Figure 2:**
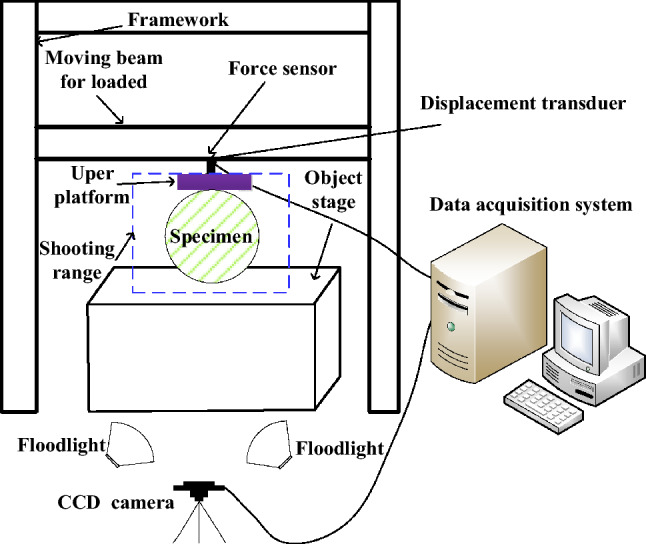
Schematic diagram of splitting tension device.

As shown in Fig. 2, the PIV test equipment includes an image capture system and a loading system. The image capture system consists of a high-speed industrial CCD camera, floodlights, and DaVis 8.0 series software. The loading system is a model CMT4000 electronic universal testing machine (Mester Company, United States) composed of loading equipment and a data acquisition system. The universal testing machine parameters can be set as required, with automatic constant speed loading. Because the linear variable differential transducer (LVDT) deformation sensor and loading device collect information simultaneously, the specimen deformation can be considered to be coordinated with the load imposed by the loading device^45,46^.

#### Microscopic test

SEM tests can qualitatively reveal the structural arrangement of particles and pores on the soil’s surface using secondary electron signal imaging. A model JSM-7600 field emission scanning electron microscope (JEOL Company, Japan) was used in this study^47^. The SEM resolution is 1.0 nm (15 kV)/1.4 nm (1 kV), the acceleration voltage range is 0.1–30 kV, and the amplification can be up to one million times.

MIP is an accurate and effective method for determining soil pore size distribution through quantitative analysis. An AutoPore 9600 instrument (Micromeritics, United States) was used in the MIP test^52^. Its measurable pore diameter ranges from 5.5 nm to 363.6 μm, and the pressure ranges from 3.4 kPa to 227.5 MPa.

### Specimen preparation and testing program

**Figure 3:**
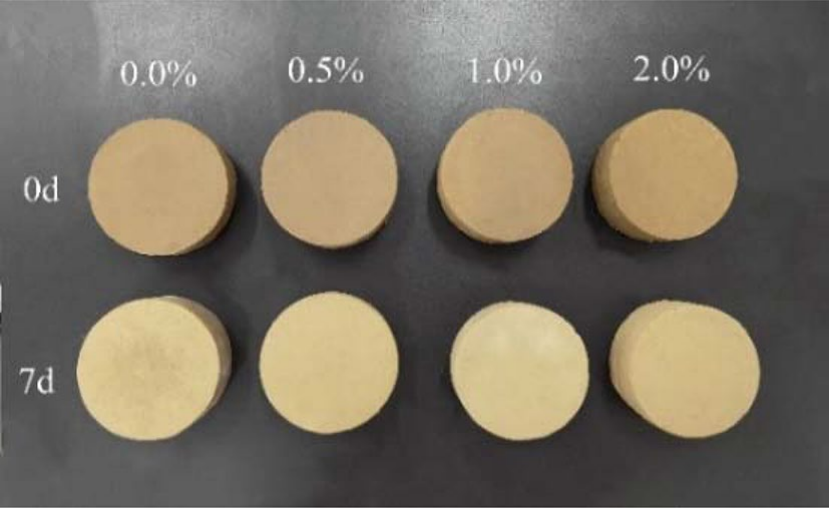
Photographs of splitting tension test specimens.

The initial water content of the specimens in all tests was designed to be 20% to realize dehydration^30,31^. The XG contents were controlled at 0.0%, 0.5%, 1.0% and 2.0% by mass ratio (*m*_b_/*m*_s_) of XG (*m*_b_) to soil (*m*_s_). The specimens were prepared by the dry mixing technique^48^, which mixed XG and loess powder evenly and then added the required quantity of water. The XG–loess mixtures were then placed in a sealed desiccator for braising for 24 h. Specimens (63.5 mm in diameter and 25.2 mm in height) were compacted in a cylindrical mold (Fig. 3). The specimen curing conditions were room temperature (25 °C), 50% relative humidity, and natural air drying.

#### Splitting tension test

The test procedure is as follows: (1) First, the ring specimens for three initial dry densities of 1.55 g/cm^3^, 1.63 g/cm^3^, and 1.70 g/cm^3^ were prepared and dried for seven days. (2) The specimens were placed on the universal testing machine, and the position of the upper pressure plate was adjusted until it contacted the top of the specimen. At the same time, it was ensured that the pressure sensor was well connected to the computer, and the collection parameters of the loading device were set according to the test requirements. Constant displacement control was selected, and the displacement descent rate was set to 1.4 mm/min. (3) The floodlights and camera were adjusted to ensure that image sharpness and field of view were optimal. (4) The total number and frequency of photos by the PIV measurement system were set to 1500 and 7 photos/s, respectively. The test concluded after the apparent failure of the specimen. (5) The pictures taken at each stage were selected according to the load–displacement curve, and the PIV analysis system was used to compare the pictures. The soil deformation field during crack development was analyzed, and the displacement vector figure was generated.

#### Microscopic test

The change in microstructure for soils with different initial dry densities is similar^53,54^. Therefore, the specimens with a dry density of 1.63 g/cm^3^ were chosen as representative for the microscopic test. Specimens with 0.0% and 2.0% XG contents were prepared and cured for seven days. The specimens were placed in liquid nitrogen for rapid cooling and then freeze-dried for 24 h to preserve the original structure of the specimens as much as possible. For the next step of the tests, the specimens were cut into small pieces. Before SEM observation, a thin layer of gold was plated on the surface of the specimens. Multiple soil points were taken for shooting, and the SEM image magnifications were 50, 100, 200, 500, 1000, 2000, and 10,000. Two parallel tests were carried out for each group of soil specimens, and the best specimen was selected for subsequent SEM and MIP tests to ensure their integrity.

## Results and discussion

### Microscopic test

#### SEM images

**Figure 4:**
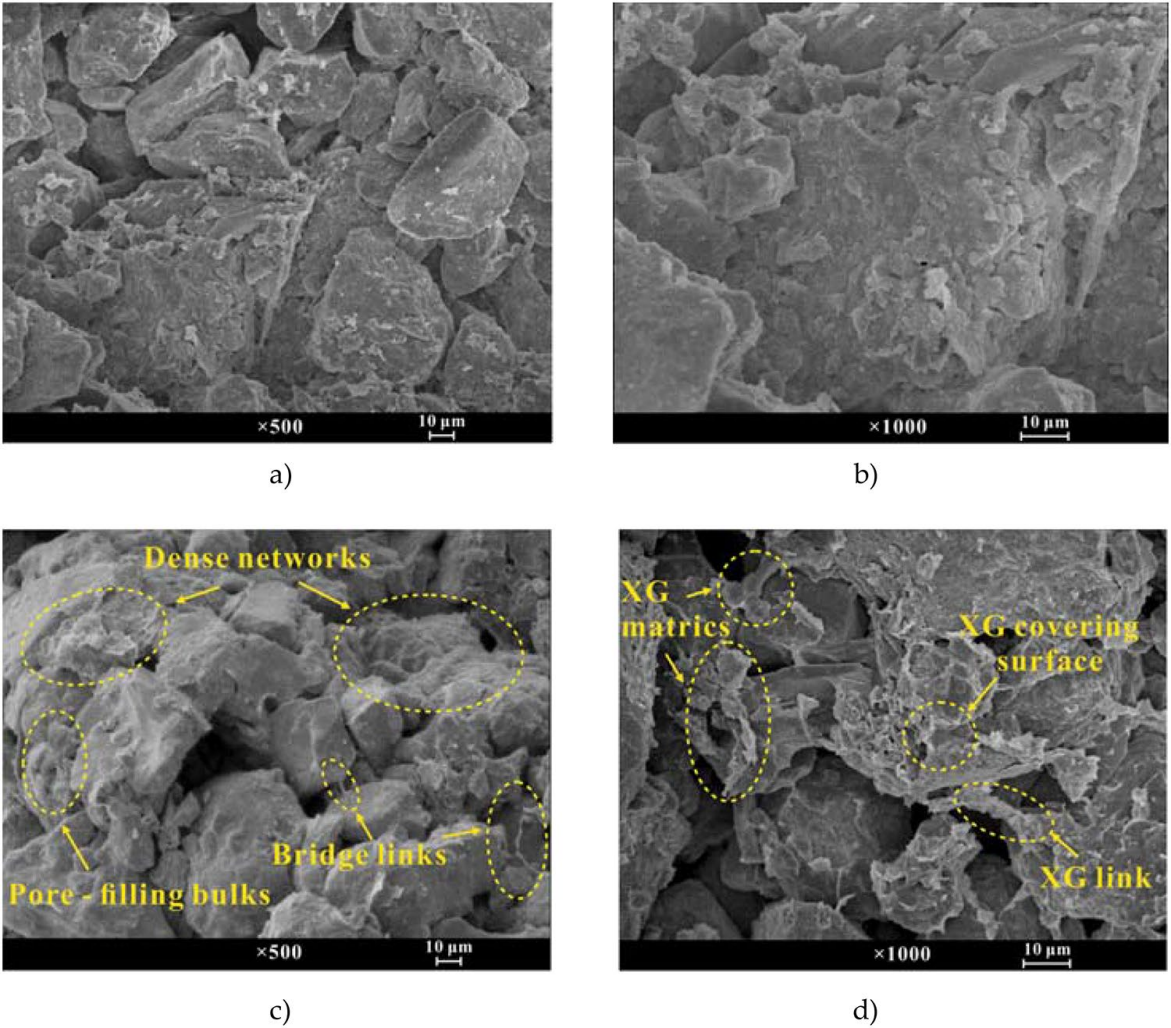
(**a**) SEM image of untreated soil at × 500; (**b**) SEM image of untreated soil at × 1000; (**c**) SEM image of the 2.0% XG-treated soil at × 500; (**d**) SEM image of the 2.0% XG-treated soil at × 1000.

The SEM images describe the microstructure with different XG content levels. The SEM images with magnifications of 500 and 1000 for soil with 0.0% XG content are shown in Fig. 4a, b. After seven days of dry curing at constant temperature and humidity, the full development of the aggregated structure gave the particles or aggregates an angular appearance. The arrangement of particles was mainly mosaic, and the adhesion came only from particle connections with point–point and face–point contact.

The SEM images at a magnification of 500 for soil with 2.0% XG content are shown in Fig. 4c. Compared with the images for the untreated soil, the addition of the XG induced the formation of flocs and created soil particle line contacts or face contacts^41^. Some of the XG filled the pore as a bulk, while others formed bridge links or dense networks to bond loess particles. As the image is enlarged in Fig. 4d, cross-linking between XG and soil can be further observed. After seven days of dry curing, the XG became short and agglomerated by precipitation and shrinkage, and the XG viscosity increased^38^. The XG initially filled in or near the pores, developing into covering surfaces, merging into bridge links, or accumulating into matrices, which connected the adjacent soil frameworks closely^23,28,29,32,48^. As the contact area between the soil particles increased, their integrity was enhanced, and the particle size of soil skeleton particles increased.

#### MIP images

**Figure 5:**
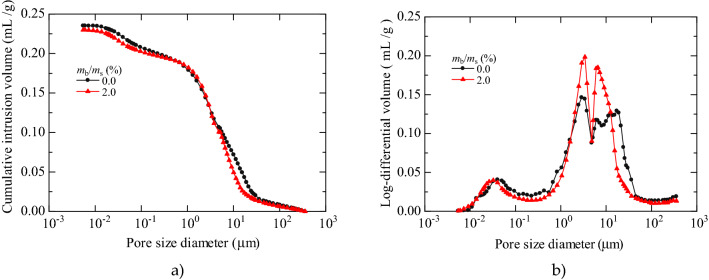
MIP test results. (**a**) Cumulative intrusion curve; (**b**) Pore size distribution density curve.

Figure 5a, b depict the cumulative mercury intrusion curve and pore size distribution (PSD) curve of specimens with 0.0% and 2.0% XG contents (*m*_b_/*m*_s_), respectively. As can be seen from Fig. 5a, the pore diameter of the specimens with 0.0% and 2.0% XG contents were mainly concentrated in the pore diameter range of 1–30 μm. The pore volumes of the two types of specimens were almost the same.

As presented in Fig. 5b, the PSD curves of two types of specimens showed multi-peak characteristics. According to previous microstructural test results^55,56^, the pores of the loess specimens were divided into ultra-micropores (< 1 μm), micropores (1–5 μm), mesopores (5–45 μm), and macropores (≥ 45 μm). The PSD curves of the untreated and XG-treated specimens were the same in the ultra-micropore and macropore ranges. However, there were differences in the concentrated micropore and mesopore distributions. With the addition of XG, the peak PSD value and corresponding pore size in the ultra-micropore range remained unchanged at 0.4 mL/g and 0.04 μm, respectively. The pore size corresponding to the peak PSD value in the micropore range remained unchanged at 3.4 μm, and the peak PSD value increased from 0.14 to 0.20 mL/g. The pore size corresponding to the peak PSD value in the mesopore range decreased from 17.3 to 7.3 μm, and the peak PSD value increased from 0.13 to 0.18 mL/g. The soil particles and XG in the XG-treated specimen fully shrink and aggregate during the drying and curing processes. Therefore, the XG-treated specimens are dominated by micropores and mesopores. The peak values of XG-treated specimens were higher than for the untreated specimens.

### Splitting tension test

#### Load–displacement curve and splitting tensile strength

**Figure 6:**
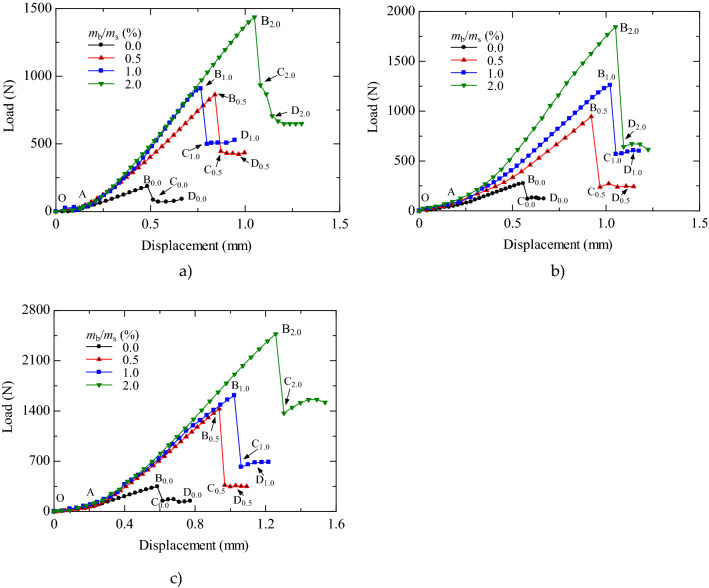
Load–displacement curves of specimens with different initial dry densities. (**a**) 1.55 g/cm^3^; (**b**) 1.63 g/cm^3^; (**c**) 1.70 g/cm^3^.

The load–displacement curves of the XG-treated specimens with initial dry densities of 1.55 g/cm^3^, 1.63 g/cm^3^, and 1.70 g/cm^3^ are presented in Fig. 6a–c. The labels (*A–D*) in Fig. 6 represent crack development feature points, and their subscripts represent the XG content.

After curing for seven days, the load–displacement curves of specimens with different XG contents showed a prominent strain-softening behavior. These curves can be divided into different stages as follows: *OA*: The slope of load-displacement curves increased gradually, and a stress concentration occurred at the contact position between the specimen and the upper and lower pressure heads; *AB*: The specimen load increased linearly with increasing displacement. At this stage, the specimen deformed elastically, and the specimen interiors tended to become compact; *BC*: The curves dropped sharply after reaching the failure load value. The specimen entered the unstable failure stage, and vertical cracks began to appear clearly on the specimen surface. *CD*: The specimen load was stable with increasing displacement. Cracks developed on the surface and inside the specimen until the specimen was destroyed^45,46^.

The load–displacement curves of specimens varied with XG content and dry density. With increased XG content, the load–displacement curve softening phenomenon became more pronounced, and the specimen became brittleness increased. Generally, a higher dry density corresponded to a more prominent softening phenomenon and a higher failure load.

**Figure 7:**
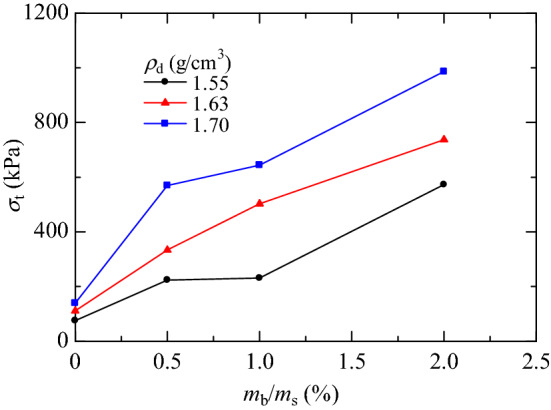
Relationship between splitting tensile strength and XG content.

The splitting tensile strength (*σ*_t_) is derived from the failure load (*P*), diameter (*d*), and height (*t*) of the specimen, as shown in Eq. (1). The failure load *P* is the load corresponding to point *B* on the load–displacement curve. From the summary table of splitting test data (Table 3), it can be seen that the water content (*w*) and saturation (*S*_r_) of the specimens increased slightly with increasing XG content. As shown in the relationship curves between splitting tensile strength and XG content with different initial dry densities (Fig. 7), all XG-treated specimens had higher splitting tensile strengths than untreated specimens^42,43^. The higher the XG content and dry density, the greater the splitting tensile strength of the specimens. The splitting tensile strengths of specimens with 2.0% XG content were the highest. At dry densities of 1.55 g/cm^3^, 1.63 g/cm^3^, and 1.70 g/cm^3^, the soil strength increased by factors of 6.58, 5.62, and 6.06, respectively.

**Table 3:** Summary of splitting tension tests data.

*ρ*_d0_ (g/cm^3^)	(*m*_b_/*m*_s_) (%)	*W *(%)	*S*_r_ (%)	*e*	*σ*_t_ (kPa)	Fracture type
1.55	0.0	1.04	4.10	0.69	75.55	I
	0.5	1.21	4.70	0.70	224.01	I
	1.0	1.33	5.13	0.70	231.39	I
	2.0	1.50	5.76	0.70	572.96	I
1.63	0.0	1.23	5.21	0.62	111.39	I
	0.5	1.24	5.30	0.63	333.88	I
	1.0	1.31	5.63	0.63	502.69	I
	2.0	1.54	6.59	0.63	737.33	II
1.70	0.0	1.16	5.55	0.56	139.63	I
	0.5	1.18	5.56	0.57	569.56	I
	1.0	1.34	6.33	0.57	644.07	I
	2.0	1.54	7.29	0.57	986.18	II

After curing for seven days, the water content of the specimens was below 2.0%, so the specimens were dehydrated, which provided the conditions for the formation of the XG matrix and mutual bonding between the XG and soil particles^29^. The dense fiber network formed by XG could reduce water loss, thereby increasing the water content and saturation. The ability of the soil to resist external forces mainly came from the biopolymer bond strength. Having a bridge connection or dense mesh formed by XG created stronger interparticle connections between the soil and XG so that the splitting tensile strength was enhanced during the dehydration process^39,41,47^.

1 $$ \sigma_{{\text{t}}} = - \frac{2P}{{\pi dt}} $$

#### Fracture propagation and displacement vector field

**Figure 8:**
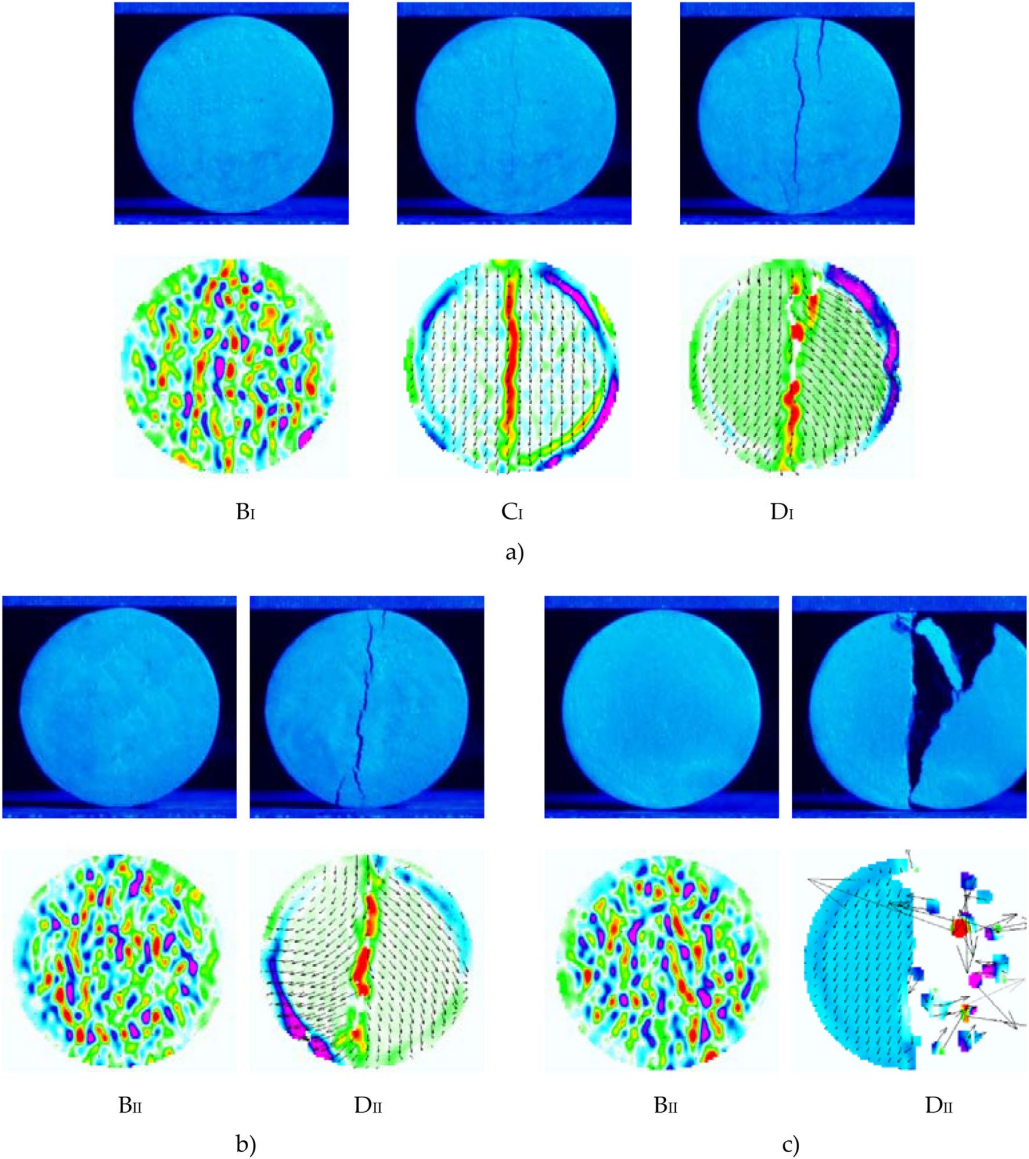
Fracture propagation and displacement vector fields of specimens at selected points (*B*–*D*) on the load–displacement curve: (**a**) Type I; (**b**) Type II for 1.63 g/cm^3^ initial dry density; (**c**) Type II for 1.70 g/cm^3^ initial dry density.

The fracture propagation and displacement vector fields obtained from the splitting tension tests can be divided into two types (I and II), as shown in Fig. 8. The darker areas in the images have more concentrated stresses, more significant deformations, and more noticeable cracks. The displacement vector fields of the two types had the same performance at point *B*. The specimen had compression deformation at point *B* without apparent cracks, and the displacement was mainly concentrated in the specimen interior. However, they were different at points *C* and *D*.

For Type I specimens, after the load drops from point *B* to point *C*, a fine crack appeared in the center of the specimen, the displacement vector field was symmetrically distributed on both sides of the splitting surface, and the specimen began to fail. At point *D*, the primary cracks were radial–vertical, and the secondary fractures were well-developed. The displacement vector field obtained by the PIV technique is a blank area for the failure part of the specimen because of its large displacement. For Type II specimens, after the failure load is reached, the specimen did not exhibit a fine crack, as shown for point *C*, but immediately had penetrating cracks when the load dropped to point *D* and then fell immediately.

The splitting types of specimens with different initial dry densities are also summarized in Table 3. The specimens’ brittle failure and radial–vertical cracks were consistent with the apparent strain-softening of the load–displacement curve. Regardless of the dry density value, the loess brittleness was effectively increased by XG treatment compared with pure loess after curing for seven days. The specimens presented as Type II had higher XG content (2.0%) and dry density (1.63 g/cm^3^ and 1.70 g/cm^3^). Higher XG content resulted in faster crack development and more brittleness for the same dry density within the studied range of XG content (0.0–2.0%). When the XG content was 2.0%, the brittleness of the specimen was the highest. This increase in brittleness was attributed to the stronger cementation and denser structure formed by cross-linking between the XG and soil at higher XG content.

## Conclusions

Splitting tensile tests were carried out on XG-treated loess with different XG contents and initial dry densities using a measurement technique based on particle image velocimetry. The microstructure of the specimens was qualitatively and quantitatively analyzed by scanning electron microscopy and mercury intrusion porosimetry. The main conclusions drawn from the study are as follows.

The XG gradually shrunk and accumulated during drying and curing, developing into covering surfaces, merging into bridge links, or accumulating into fiber matrices, forming a dense network closely connecting the adjacent soil frameworks. The micropores and mesopores mainly existed in the specimens treated with XG. The peak values of the PSD curves for XG-treated specimens were higher than those of untreated specimens.

The XG was found to be effective for stabilizing loess. After drying for seven days, all the specimens’ load–displacement curves demonstrated strain-softening behavior. The splitting tensile strength increased nonlinearly with increasing XG content for different initial dry densities. Adding XG increased the splitting tensile strength of loess by about a factor of 6 times. The specimens with higher XG content had a higher water content and saturation. The fiber-interconnected network formed by XG prevented water loss and enhanced the splitting tensile strength.

In the splitting tensile test, the fracture propagation and displacement vector field had an apparent one-to-one correspondence with different stages of the load–displacement curve. The compression deformation, initial cracking, crack development, and penetrating failure stages could all be observed using the PIV technology. The specimens presented radial–vertical primary and relatively developed secondary cracks. The soil structure became denser and more flocculated with increasing XG content and dry density, leading to an increase in the brittleness of the specimens. The penetrating cracks appeared immediately after the peak splitting tensile strength for the specimens with 2.0% XG content and initial dry densities of 1.63 g/cm^3^ and 1.70 g/cm^3^.

XG is an eco-friendly and renewable material with great potential in loess stabilization applications. This research on XG-treated loess provides a new way to improve soil strength and guarantee safe operation during infrastructure construction projects (such as building foundations, dams, highways, slopes, tunnels) in loess areas.
